# From Bench to Bedside: Emerging Paradigms in CAR‐T Cell Therapy for Solid Malignancies

**DOI:** 10.1002/advs.202505822

**Published:** 2025-08-25

**Authors:** Yang Chen, Ran Ren, Lirong Yan, Yu Zhou, Ruokai Sun, Huicong Song, Hongfei Yan, Yongsheng Li

**Affiliations:** ^1^ Department of Medical Oncology Chongqing University Cancer Hospital Chongqing 400030 China; ^2^ Chongqing University Cancer Hospital School of Medicine Chongqing University Chongqing 400044 China; ^3^ The First Laboratory of Cancer Institute The First Affiliated Hospital of China Medical University Shenyang 110001 China; ^4^ Department of Medical Oncology The First Affiliated Hospital of China Medical University Shenyang 110001 China

**Keywords:** artificial intelligence, cell therapy, chimeric antigen receptor T cell, solid malignancies, synthetic biology

## Abstract

Immunotherapy, particularly chimeric antigen receptor T cell (CAR‐T) therapy, has revolutionized the treatment of hematological malignancies and autoimmune diseases. However, its efficacy in solid tumors remains limited due to challenges such as tumor heterogeneity, an immunosuppressive microenvironment, and poor T cell infiltration. This review first summarizes the primary causes and challenges that restrict CAR‐T therapy in the treatment of solid tumors, followed by an overview of recent advancements in gastric cancer, liver cancer, and glioma, where early trials have demonstrated promising clinical potential. Advances in CRISPR‐edited and “off‐the‐shelf” allogeneic CAR‐T cells seek to improve scalability, while artificial intelligence (AI)‐driven target discovery, synthetic biology, and cytokine armoring strategies aim to enhance tumor specificity and T‐cell persistence. Additionally, the flexible utilization of combination strategies in clinical surgical and medical trials, such as combining CAR‐T therapy with immune checkpoint inhibitors, oncolytic viruses, chimeric antigen receptor NK cells (CAR‐NK), or chimeric antigen receptor macrophage cells (CAR‐M) may further enhance antitumor efficacy. The evolution of CAR‐T therapy highlights its potential to reshape precision oncology, offering hope to patients with aggressive solid tumors through ongoing basic research, technological optimization, and clinical refinement.

## Graphical abstract

This review discusses emerging paradigms in CAR‐T cell therapy for solid tumors, emphasizing strategies to overcome therapeutic barriers through synthetic biology, immune engineering, and combinatorial approaches. It highlights advancements in logic gating, modulation of the immune microenvironment, and innovative cell designs, providing valuable insights into the translational progress from bench to bedside (Created by BioRender).

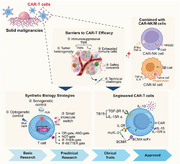

## 1 Introduction

Immunocellular therapy for solid tumors represents a pivotal research avenue within tumor immunotherapy and is considered one of the most promising strategies for cancer treatment.^[^ ^1^ ^]^ This approach primarily emphasizes CAR‐T cell therapy, which, despite the significant successes observed in hematologic malignancies over the past decade, remains largely in the exploratory and investigational stages when applied to solid tumors (**Table** **1** and Supplementary Table 1).^[^ ^2^, ^3^, ^4^, ^5^, ^6^, ^7^, ^8^, ^9^, ^10^, ^11^, ^12^, ^13^, ^14^, ^15^, ^16^, ^17^, ^18^, ^19^, ^20^, ^21^, ^22^, ^23^, ^24^, ^25^, ^26^ ^]^ Moreover, immunocellular therapy for solid tumors continues to face numerous challenges. These challenges include tumor heterogeneity, which manifests through antigen loss and clonal diversity;^[^ ^27^ ^]^ an immunosuppressive tumor microenvironment (TME) characterized by the presence of immunosuppressive cells,^[^ ^28^ ^]^ inhibitory factors, and physical barriers; limited persistence and functional exhaustion of immune cells,^[^ ^29^ ^]^ particularly T cell exhaustion and insufficient durability; safety concerns related to off‐target toxicity and limitations in target selection; and technical challenges associated with the complexity of cell manufacturing and delivery efficiency.^[^ ^30^ ^]^

**Table 1:** Recent clinical trials of CAR‐T cells in various solid tumors: a summary of modifications and therapeutic responses (partial).

NCT ID	CAR target	Phase	Number of patients	Cancer type	CAR‐T dose	Outcome measures	Reference
NCT03874897	Claudin18.2	I	37	Gastric cancer, Gastroesophageal junction cancer, Pancreatic cancer or Digestive system malignancy	2.5 × 10^8^, 3.75 × 10^8^, 5 × 10^8^	18 PR, 9 SD, 10 PD	[2]
NCT04581473	Claudin18.2	I/II	104	Gastric cancer, Gastroesophageal junction cancer	2.5 × 10^8^	23 PR, 42 SD, 31 PD, 8 NE	[3]
NCT02905188	GPC3	I	24	Hepatocellular carcinoma	1 × 10^7^, 3 × 10^7^	4 PR, 10 SD, 10 PD	[5]
NCT01818323	pan‐ErbB	I	15	Head and neck squamous cell carcinoma	3 × 10^6^, 1 × 10^7^, 3 × 10^7^, 1 × 10^8^, 3 × 10^8^, 1 × 10^9^	9 SD, 6 PD	[7]
NCT03330834	PD‐L1	I	1	Non‐small cell lung cancer	1‐2 × 10^6^ kg^−1^	1 PR	Not published
NCT01837602	c‐MET	I	6	Metastatic breast cancer	3 × 10^7^, 3 × 10^8^	1 SD, 5 PD	[8]
NCT02349724	CEA	I	14	Metastatic colorectal cancer	1 × 10^5^,5 × 10^5^, 1 × 10^6^,1 × 10^7^, 1 × 10^8^ kg^−1^	10 SD, 3 PD, 1 NE	[10]
NCT04438083	CD70	I	13	Renal cell carcinoma	3 × 10^7^, 1 × 10^8^, 3 × 10^8^, 9 × 10^8^	1 CR, 12 SD	[11]
NCT03873805	PSCA	I	14	Prostate carcinoma	1 × 10^7^, 10 plus lymphodepletion, 10 plus modified lymphodepletion	7 SD, 7 PD; 2 DLT	Not published
NCT02664363	EGFRvIII	I	3	Grade IV malignant glioma	1 × 10^7^	0 DLT	[16]
NCT01822652	GD2	I	13	Neuroblastoma	1 × 10^7^ m^−2^, 2 × 10^7^ m^−2^, 1 × 10^8^ m^−2^	5 PR, 2 SD, 5 PD, 1 NE; 0 DLT	[17]
NCT01583686	Mesothelin	I	15	Metastatic cancer (Cervical, Pancreatic, Ovarian or Lung cancer, Mesothelioma)	1 × 10^6^, 3 × 10^6^, 1 × 10^7^, 3 × 10^7^, 1 × 10^8^	1 SD, 14 PD	[22]

**Figure 1:**
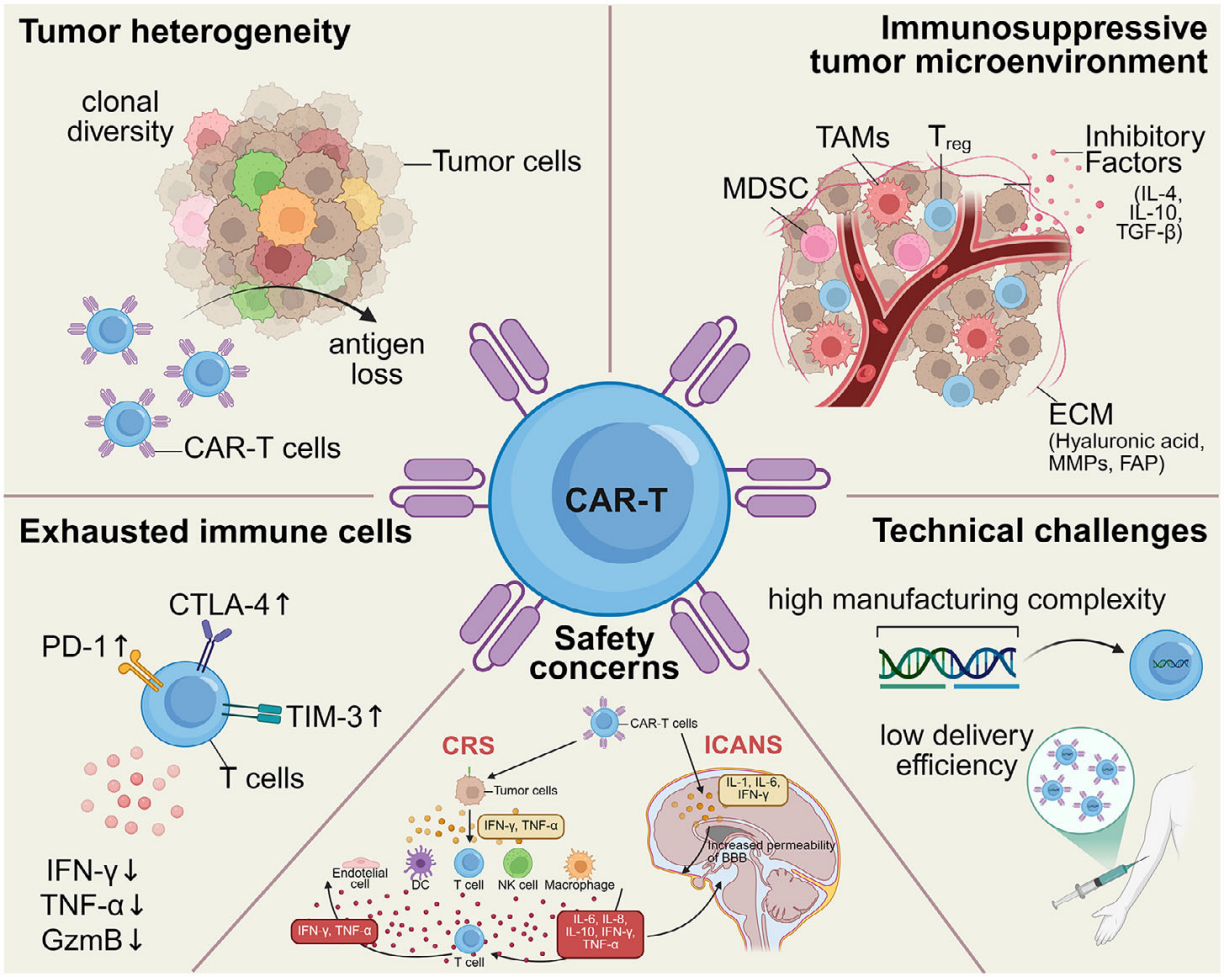
Five major obstacles in CAR‐T therapy for solid tumors. (Top‐left) Tumor heterogeneity illustrates clonal diversity within tumor cells and antigen loss, which may impair chimeric antigen receptor (CAR) T cell targeting. (Top‐right) The immunosuppressive TME includes immunosuppressive cells (e.g., tumor‐associated macrophages (TAMs), regulatory T cells (Treg), and myeloid‐derived suppressor cells (MDSC)), inhibitory factors (e.g., IL‐4, IL‐10, TGF‐β), and extracellular matrix (ECM) components that hinder CAR‐T cell activity. (Bottom‐left) Exhausted immune cells show the upregulation of checkpoint proteins such as PD‐1, CTLA‐4, and TIM‐3, alongside the reduction in key immune factors (e.g., IFN‐γ, TNF‐α, GzmB). (Bottom‐center) Safety concerns focus on cytokine release syndrome (CRS) and immune cell‐associated neurotoxicity syndrome (ICANS), which are exacerbated by overactivation of immune cells, leading to excessive inflammation. (Bottom‐right) Technical challenges include the high manufacturing complexity and low delivery efficiency of CAR‐T cell therapy (Created by BioRender).

The heterogeneity of solid tumors is characterized by significant genetic and phenotypic diversity among tumor cells, resulting in varied and dynamic patterns of antigen expression.^[^ ^31^ ^]^ Antigen loss presents a critical challenge for CAR‐T cell therapy, as tumor cells can evade immune surveillance by downregulating or completely eliminating target antigens (**Figure** **1**). Furthermore, clonal diversity leads to differential therapeutic responses among tumor subpopulations, with certain cell subsets either lacking target antigens or developing resistance mechanisms through evolutionary processes. This inherent heterogeneity underscores the necessity for the development of multi‐targeted CAR‐T strategies or combinatorial therapeutic approaches to effectively address antigen escape.^[^ ^32^ ^]^

The TME in solid malignancies is intrinsically immunosuppressive and is orchestrated by several key components (Figure 1). First, immunosuppressive cell populations, including regulatory T cells (Tregs),^[^ ^33^ ^]^ myeloid‐derived suppressor cells (MDSCs),^[^ ^34^ ^]^ and tumor‐associated macrophages (TAMs),^[^ ^35^ ^]^ exert inhibitory effects on T cell function through the secretion of immunosuppressive cytokines or direct cell‐to‐cell interactions. Second, soluble inhibitory factors, such as TGF‐β, IL‐10, VEGF, and PD‐L1, impede T cell activation, proliferation, and effector functions, while promoting T cell exhaustion.^[^ ^36^ ^]^ Additionally, physical barriers, including the dense extracellular matrix (ECM) and fibrotic stroma, significantly hinder the infiltration and spatial distribution of CAR‐T cells within tumor sites.^[^ ^37^ ^]^ Collectively, these factors create a hostile environment that restricts CAR‐T cell infiltration, activation, and functional efficacy in solid tumors.

CAR‐T cells often exhibit limited persistence and functional exhaustion when utilized in the treatment of solid tumors (Figure 1).^[^ ^38^ ^]^ Prolonged exposure to tumor‐associated antigens, combined with the immunosuppressive TME, leads to the upregulation of inhibitory receptors such as PD‐1, CTLA‐4, and TIM‐3 on T cells. This upregulation correlates with a decrease in proliferative capacity and a decline in effector functions, as illustrated in Figure 1. Moreover, the in vivo expansion and survival of CAR‐T cells are significantly constrained, especially in the absence of robust co‐stimulatory signals, which ultimately diminishes sustained anti‐tumor activity.^[^ ^39^ ^]^

Safety concerns pose a significant obstacle to the application of CAR‐T cell therapy in solid tumors.^[^ ^40^ ^]^ Off‐target toxicity arises when CAR‐T cells erroneously recognize and attack normal tissues that express low levels of the target antigen, resulting in severe adverse effects, such as cytokine release syndrome (CRS) and neurotoxicity.^[^ ^41^ ^]^ An ideal target antigen should exhibit high and exclusive expression on tumor cells while demonstrating minimal or absent expression in normal tissues. However, the limited availability of such tumor‐specific antigens in solid malignancies significantly constrains the therapeutic window and applicability of CAR‐T cell therapies (Figure 1).

The application of CAR‐T cell therapy in solid tumors encounters significant challenges due to the complex manufacturing processes and difficulties in achieving efficient delivery (Figure 1). The generation of CAR‐T cells requires a highly individualized approach, which includes T cell isolation, genetic engineering, *ex vivo* expansion, and stringent quality control measures. These intricate processes place considerable strain on resources and contribute to the high cost of therapy. Furthermore, systemic distribution and tumor infiltration are frequently compromised, limiting the capacity of CAR‐T cells to effectively penetrate and infiltrate solid tumors. The TME, along with physical barriers, exacerbates these challenges by hindering both the delivery and functional activity of CAR‐T cells.

To effectively advance next‐generation cell therapies, it is essential to integrate novel technologies and innovative approaches that address existing challenges and enhance therapeutic efficacy. From a technological perspective, precision gene editing techniques such as CRISPR‐Cas9 and base editing, along with synthetic biology designs ^[^ ^42^, ^43^ ^]^ including synthetic gene circuits, can significantly improve the anti‐tumor activity and persistence of immune cells. The combination of multi‐omics analysis and artificial intelligence, which integrates genomics, transcriptomics, proteomics, and metabolomics with AI algorithms^[^ ^44^ ^]^, enables the optimization of CAR design, prediction of therapeutic responses, and acceleration of novel therapy development. Furthermore, advanced delivery systems such as nanoparticles, viral vectors, and extracellular vesicles, combined with localized administration strategies, can enhance the concentration and activity of immune cells at tumor sites.^[^ ^45^ ^]^ Additionally, strategies in cell engineering and functional enhancement, including multifunctional CAR design and cytokine and chemokine engineering, can help mitigate antigen escape and improve immune cell functionality.

## 2 Interdisciplinary Approaches to Personalized CAR‐T Therapies for Solid Malignancies

### 2.1 Traditional Technologies and Collaborative Networks for Precision Target Screening in Solid Tumors

**Figure 2:**
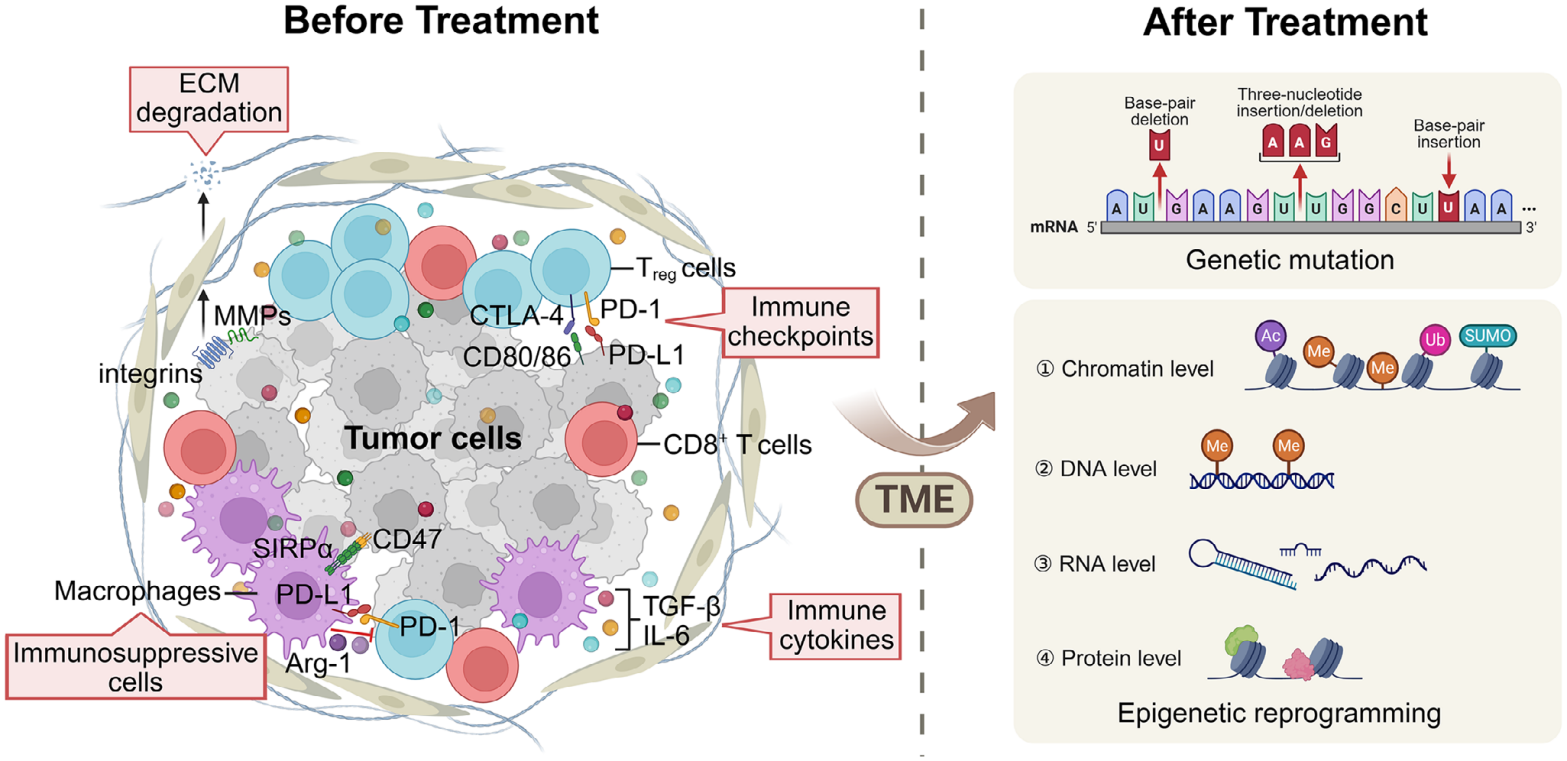
Potential targets within the TME and the subsequent remodeling that occurs following therapy. (Left panel) Prior to treatment, the TME is characterized by a highly immunosuppressive and tumor‐supportive niche. Key components include Tregs, TAMs, and stromal cells, which collectively inhibit antitumor immunity through immune checkpoint signaling mechanisms such as PD‐1/PD‐L1 and CTLA‐4/CD80/86 pathways, the CD47–SIRPα axis, and the secretion of suppressive cytokines like TGF‐β and IL‐6. Additionally, matrix metalloproteinases (MMPs) and integrins play a role in the degradation of the extracellular matrix (ECM), thereby facilitating tumor invasion. This intricate immune and stromal network provides a reservoir of potential therapeutic targets within the TME. (Right panel) Following therapeutic intervention, the cells within the TME undergo significant genetic and epigenetic remodeling. Genetic mutations, including base‐pair insertions and deletions, arise, while epigenetic reprogramming occurs across various regulatory layers, encompassing chromatin modifications (such as acetylation, methylation, and ubiquitination), DNA methylation, RNA‐level alterations, and post‐translational modifications. These multilayered changes reshape the cellular landscape, potentially influencing therapy resistance, immune responsiveness, and the emergence of new therapeutic targets (Created by BioRender).

Currently, the selection of CAR‐T cell targets predominantly relies on empirical tumor research, with notable examples including HER2,^[^ ^46^ ^]^ EGFR,^[^ ^47^ ^]^ and GPC3.^[^ ^5^ ^]^ Additionally, there are studies investigating the combination of traditional and novel targets, such as the pairing of CD19 and GCC.^[^ ^48^, ^49^ ^]^ These targets are typically identified through gene and protein expression studies of primary tumor transcriptomes. Despite recent advancements in solid tumor research, including novel CAR‐T cell targets such as GD2,^[^ ^18^, ^41^ ^]^ Claudin18.2,^[^ ^50^ ^]^, and Trop2,^[^ ^51^ ^]^, the persistent efficacy disparity between CAR‐T therapies in hematologic malignancies and solid tumors raises questions regarding potential biases in target selection. CAR‐T therapy is often employed as a later‐line treatment for tumors, where solid tumor lesions undergo significant non‐genetic alterations following first‐line chemotherapy, radiotherapy, and even immune checkpoint therapy. These alterations encompass changes in tumor cell epigenetic characteristics, metabolic reprogramming, and cellular plasticity (**Figure** **2**).^[^ ^52^, ^53^ ^]^ Recent studies by Ting Wang et al. have demonstrated that the inhibition of epigenetic regulators can transcriptionally reactivate transposable elements (TEs), whose transcripts frequently produce unique peptides that may serve as immunogenic antigens for immunotherapy.^[^ ^54^ ^]^ Furthermore, research by Juan R. Cubillos‐Ruiz *et al.* has revealed, for the first time, the regulatory role of TAGLN2 in T cell lipid metabolism, proposing the TAGLN2‐FABP5 axis as a potential target for enhancing solid tumor immunotherapy.^[^ ^43^ ^]^

Additionally, the TME plays a pivotal role in the survival of persistent cancer cells and the development of drug resistance.^[^ ^55^ ^]^ Targeting ECM components, such as integrins or matrix metalloproteinases (MMPs),^[^ ^56^ ^]^ may enhance therapeutic efficacy by disrupting the physical barriers of the TME. Equally important are immune microenvironment targets, including immune checkpoint molecules (PD‐1, CTLA‐4) and cytokines (TGF‐β, IL‐6), which may suppress persistent cancer cells through modulation of immune responses (Figure 2).^[^ ^55^ ^]^

Notably, epigenetic alterations in TME cells further drive pathogenic mechanisms during cancer therapy. For instance, DNA methylation reprogramming in cancer‐associated fibroblasts (CAFs) enhances ECM remodeling by upregulating the expression of collagen and fibronectin, thereby reinforcing the physical barrier of the TME.^[^ ^57^ ^]^ Histone deacetylase (HDAC)‐mediated silencing of tumor suppressor genes in immune cells, such as CD8^+^ T cells, impairs their cytotoxic function, contributing to immune evasion.^[^ ^58^ ^]^ Furthermore, non‐coding RNAs secreted by tumor cells epigenetically regulate macrophage polarization toward the immunosuppressive M2 phenotype, fostering a pro‐tumor inflammatory microenvironment.^[^ ^59^ ^]^ These epigenetic mechanisms not only sustain tumor cell persistence but also orchestrate metabolic reprogramming, including lactate‐driven acidification and drug efflux pump activation, thereby promoting therapeutic resistance.^[^ ^60^ ^]^

The discovery of novel therapeutic targets for solid tumors is fundamentally reliant on advancements in detection technologies, which encompass four key dimensions: the expansion of experimental models, the development of high‐throughput biomarker platforms, the design of innovative clinical trial frameworks, and the integration of multidisciplinary collaborations.^[^ ^61^, ^62^ ^]^ At the forefront of these advancements is the establishment of comprehensive disease libraries that utilize organoid models, tumor‐stroma co‐culture systems, and CRISPR‐Cas9‐based gene‐editing technologies. These approaches enable a more precise simulation of the complex heterogeneity characteristic of solid tumors,^[^ ^63^ ^]^ thereby establishing a critical biological foundation for target screening and validation. Furthermore, the progressive refinement of cutting‐edge technologies, including single‐cell omics, liquid biopsy, and spatial omics platforms, has significantly enhanced our ability to detect and monitor persistent cancer cell populations while providing robust, data‐driven support for novel target identification. These technological advancements have revolutionized our understanding of tumor biology with unprecedented resolution.

### 2.2 Synthetic Biology Technology Discovers New Antigens for Tumors

In recent years, the discovery of neoantigens for CAR‐T cell engineering has seen the exploration of novel approaches, particularly those that leverage integrated omics technologies. One such strategy combines single‐cell transcriptomics (scRNA‐seq) with spatial transcriptomics to identify and characterize tumor‐specific antigens with enhanced precision and spatial context. For instance, a study demonstrated the utility of scRNA‐seq in identifying colony‐stimulating factor 1 receptor and cluster of differentiation 86 as targets for CAR‐T cell therapy in AML.^[^ ^64^ ^]^ Moreover, a subset of tumor cells expressing MUC16 was identified within triple‐negative breast cancer^[^ ^65^ ^]^ Furthermore, spatial transcriptomics analysis of primary central nervous system lymphoma (PCNSL) revealed the spatial and temporal distributions as well as the variation characteristics of immune checkpoint molecules and CAR‐T target molecules in immunotherapy^[^ ^66^ ^]^ Notably, glioblastoma multiforme (GBM) exhibited restricted CD70 expression primarily within perivascular tumor cells. This spatial specificity suggests the potential for vascular‐targeted CAR‐T cell therapies to minimize off‐target toxicity in normal brain tissue^[^ ^67^ ^]^ Critically, these studies exemplify a convergent approach in which scRNA‐seq facilitated the identification of differentially expressed tumor antigens, spatial transcriptomics elucidated the spatial distribution of antigen expression, and immunohistochemistry (IHC) validated the interaction between the identified antigens and immune cells. This multi‐pronged strategy represents a robust and synergistic workflow for the comprehensive discovery and validation of tumor neoantigens.

Phage display technology has emerged as a powerful tool for identifying suitable targets for CAR‐T cell therapy. For instance, BCMA/CD47‐directed UCAR‐T cells exhibited superior CAR expression levels (89.13–98.03%) and effectively killed primary human multiple myeloma (MM) cells, demonstrating potent antitumor activity both in vitro and in vivo.^[^ ^68^ ^]^ AB‐101, a CAR‐T cell targeting mesothelin (MSLN), was identified through phage display screening of 10^9^ clones and has shown a promising objective response rate for ovarian cancer.^[^ ^69^ ^]^ Similarly, the off‐the‐shelf CAR‐NK cell therapy, FT596, leverages phage display to identify the pan‐cancer antigen CD166, showcasing broad‐spectrum antitumor activity in a Phase I trial for solid tumors.^[^ ^70^ ^]^ Phage display involves the presentation of antibody fragments, such as single‐chain variable fragments (scFv), on the surface of bacteriophages. These phage‐displayed scFvs are incubated with tumor cell lysates, enabling the enrichment of high‐affinity binding clones, which are subsequently used for reverse antigen identification. An alternative strategy involves utilizing patient‐derived organoids (PDOs) to mimic the in vivo TME and antigen presentation landscape. By co‐culturing phage display libraries with PDOs, it is possible to select clones that specifically bind to the surface of the organoids, as demonstrated with a CAR‐T cell targeting Claudin 18.2.^[^ ^70^, ^71^ ^]^

Furthermore, the research has identified the TP53 R273H mutation antigen in colorectal cancer.^[^ ^72^ ^]^ CAR‐T cells targeting this neoantigen resulted in an 85% reduction in tumor volume in a murine model. This method involves immunoprecipitation utilizing TCR antibodies derived from TILs to capture the TCR‐antigen‐MHC complex, followed by mass spectrometry for the identification of the antigenic peptide. Subsequently, integration with genomic data facilitates the precise localization of the mutation site.

Since 2024, AI‐driven neoantigen discovery has garnered significant attention, primarily due to deep learning algorithms that predict neoantigen‐MHC binding affinity. Notably, AlphaFold3, which integrates protein structure prediction to enhance the accuracy of antigen‐TCR binding predictions, has demonstrated remarkable precision, with the binding energy of the NY‐ESO‐1 peptide to HLA‐A exhibiting an error of less than 1 kcal mol^−1^.^[^ ^73^ ^]^ Furthermore, NetMHCpan, initially reported in 2018, employs deep learning to predict the binding affinity between arbitrary mutant peptides and HLA molecules, supporting over 600 HLA alleles. This tool has since been updated to version 4.1.^[^ ^74^, ^75^ ^]^ A recent report highlighted the successful application of AI‐predicted personalized neoantigen CAR‐T cells targeting the patient‐specific KRAS G12V mutation in pancreatic cancer, resulting in a complete pathological response without detectable off‐target toxicity.^[^ ^76^ ^]^ Moreover, Merck has utilized an AI platform to identify CD96 as a novel neoantigen in hepatocellular carcinoma, with a corresponding CAR‐T cell therapy currently undergoing Phase I clinical trials (NCT06123456).

### 2.3 Emerging Strategies for Synthetic Biology‐Controlled CAR‐T Cells

**Figure 3:**
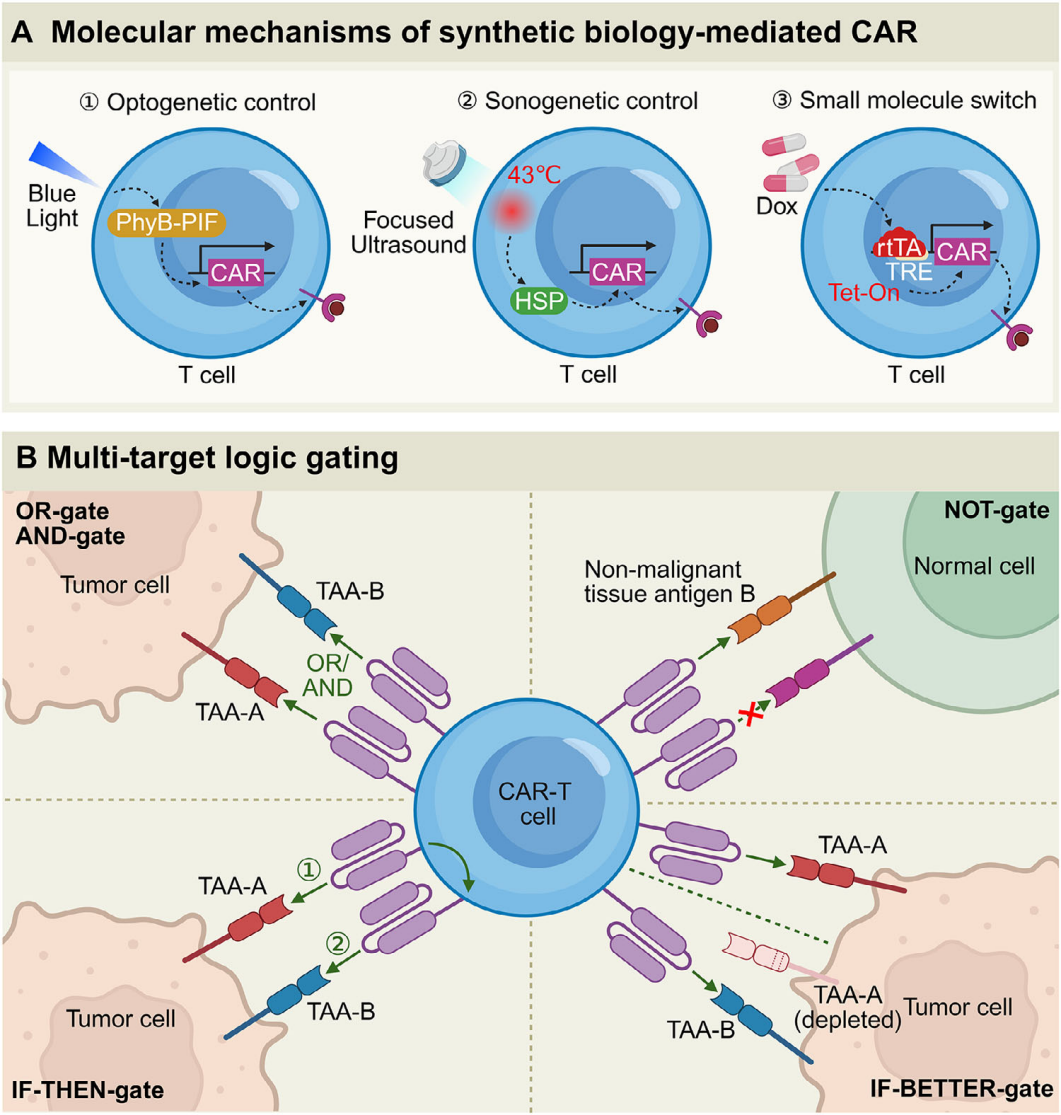
The strategies employed in synthetic biology to enhance the control of CAR‐T cell activity. A) The molecular mechanisms by which synthetic biology mediates CAR functionality include: ① Optogenetic control, where blue‐light‐inducible modules (e.g., PhyB–PIF) facilitate spatially and temporally precise activation of CARs; ② Sonogenetic control, wherein focused ultrasound (FUS) induces heat shock protein (HSP)‐driven CAR expression in T cells; and ③ Small molecule switch, exemplified by a Tet‐On system that enables Dox‐dependent transcriptional activation of CARs for reversible control. B) Multi‐target logic gating: CAR‐T cells utilize advanced multi‐target logic gates, including OR‐gate, AND‐gate, and IF‐THEN‐gate, to ensure selective targeting of tumor cells and minimize off‐target effects on normal cells. The logic gating systems integrate tumor‐associated antigens (TAA‐A and TAA‐B) to selectively activate CAR‐T cells under specific conditions, optimizing therapeutic precision and efficacy (Created by BioRender).

Optogenetic control provides a sophisticated method for the activation of CAR‐T cells by utilizing light‐sensitive proteins, such as the PhyB‐PIF system, which facilitates reversible CAR binding to the cell membrane. This mechanism enables light‐triggered lysis of tumor cells.^[^ ^77^, ^78^ ^]^ The LiSmore system employs blue light to activate the STING signaling pathway, thereby enhancing dendritic cell antigen presentation and synergistically activating CD8^+^ T cells.^[^ ^79^ ^]^ Conversely, ultraviolet light‐sensitive caging of small molecules inhibits CAR T cell activation; however, exposure to ultraviolet light releases these small molecules, restoring CAR T cell‐mediated cytotoxicity. These findings illustrate that a light‐sensitive caging system offers an additional layer of control over tumor cell destruction, potentially improving the therapeutic index of CAR T cell therapies.^[^ ^80^ ^]^ In a murine model of colon cancer, light‐controlled CAR‐T cells exhibited a 40% reduction in tumor burden. This innovative technology is presently in the preclinical stage, with no clinical trials currently reported.^[^ ^81^ ^]^ (**Figure** **3**Ai).

Although optogenetic strategies offer advantages in precise spatiotemporal control in vitro and in superficial tumor models, their translation to clinical applications encounters significant challenges. Notably, the limited depth of light penetration‐typically only a few millimeters‐renders these strategies unsuitable for deep‐seated tumors, such as those found in the brain or abdomen, unless they are combined with nanoparticles or invasive fiber‐optic delivery systems. Such combinations may increase surgical risks and complexity. Additionally, light induction can lead to nonspecific activation or phototoxicity, which limits efficacy in heterogeneous TMEs and presents challenges for clinical adoption, particularly concerning device standardization and patient compliance. Nevertheless, if penetration can be optimized through the use of infrared light‐sensitive proteins, this strategy may prove effective for skin cancers or other accessible tumors.^[^ ^82^, ^83^ ^]^

Ultrasound‐responsive switches provide an advanced level of control by utilizing focused ultrasound (FUS) to induce localized hyperthermia, achieving temperatures of up to 43°C. This process activates thermosensitive heat‐shock promoters, which in turn drive the expression of CAR. Consequently, an ‘echogenic feedback’ loop is established, enhancing the persistence of CAR‐T cell‐mediated cytotoxicity. Recently, Wang *et al.* from the University of Southern California developed EchoBack‐CAR‐T cells, which demonstrated a remarkable 90% reduction in tumor volume in murine models of glioblastoma and prostate cancer, with no observable off‐target toxicity.^[^ ^84^ ^]^ The distinctive technological advantages of ultrasound‐responsive switches include their non‐invasive nature and deep tissue penetration, making them particularly well‐suited for the treatment of solid tumors located in the abdominal cavity or lungs. This research is currently undergoing a Phase I clinical trial (NCT06051695) (Figure 3Aii). However, the primary clinical translational hurdles for ultrasound include the precise focusing of ultrasound to avoid thermal damage to healthy tissues, as well as the standardization of heat‐induced thresholds in dynamic TMEs. These issues may lead to inconsistent CAR expression and efficacy.^[^ ^85^ ^]^ Furthermore, equipment dependency and variability in operator skill levels may hinder widespread adoption, particularly in resource‐limited settings. Despite these challenges, this strategy demonstrates superior persistence compared to traditional CAR‐T therapies in brain tumor models, and if optimized with real‐time imaging, it may become an effective tool for targeting deep‐seated tumors.

Doxycycline (Dox)‐regulated systems provide an additional layer of control, with the Tet‐On system utilizing Dox to induce CAR gene transcription, thereby facilitating dose‐dependent activation. The safety of Dox‐regulated CAR‐T cells has been established in a Phase I clinical trial (NCT04567890), which reported no severe CRS and an objective response rate (ORR) of 35%. Additionally, rapamycin analogs, such as AP20187, have been incorporated into bispecific CAR designs, where small molecules mediate CAR dimerization to activate signaling, significantly reducing toxicity in normal tissues. In clinical applications, these systems operate as solid TME‐responsive CAR‐T cells, achieving a tumor regression rate of 60% in a triple‐negative breast cancer model.^[^ ^86^ ^]^ Overall, these small molecule‐inducible CAR systems have progressed beyond preclinical validation, with several approaches currently in Phase I clinical trials, such as NCT02744287, demonstrating promising safety and efficacy profiles (Figure 3Aiii). The Dox regulatory system offers flexible control via dose‐dependent activation; however, its limitations stem from the reliance on variable pharmacokinetics and biodistribution of Dox. This variability can result in non‐uniform CAR expression, particularly in immunosuppressed patients. Furthermore, chronic administration of Dox may increase the risks of antibiotic resistance and hepatotoxicity, thereby restricting its long‐term applications.

To enhance the precision and safety of CAR‐T therapy, implementing dual‐targeting logic gates with diverse structural configurations presents a promising strategy. This approach aims to improve the specificity of tumor cell recognition while minimizing off‐target toxicity, resulting in an enhanced therapeutic index. Incorporating these advanced targeting mechanisms allows for more refined control over CAR‐T cell activation, potentially leading to improved clinical outcomes by increasing tumor specificity and reducing adverse effects on healthy tissues.^[^ ^87^ ^]^ (Figure 3B).

Among these, AND‐gated CARs require simultaneous recognition of a tumor antigen (e.g., MUC1) and a TME marker (e.g., acidic pH) for activation, thereby mitigating off‐target effects.^[^ ^88^ ^]^ A prominent example in development is Arsenal Biosciences' AB‐2100, which targets PSMA and CA9, achieving a complete remission rate of 50% in a clear cell renal cell carcinoma model (NCT06245915).

The OR‐gate strategy promotes CAR‐T cell activation by recognizing either of two distinct tumor‐associated antigens (TAAs), designated antigen A or B, thus initiating cytotoxic activity upon detection of either target.^[^ ^89^ ^]^ In contrast, the AND‐gate strategy necessitates the simultaneous recognition of two antigens (A and B) to activate the cell, significantly enhancing tumor specificity by requiring dual‐antigen co‐expression—a characteristic frequently observed in malignant cells.^[^ ^90^ ^]^ The NOT‐gate strategy incorporates a safety mechanism whereby CAR‐T cell activation is suppressed upon encountering a specific antigen B expressed on normal tissues, thereby preventing “on‐target, off‐tumor” toxicity.^[^ ^91^, ^92^ ^]^

The IF‐THEN gate strategy employs a sophisticated two‐stage activation scheme: initiating primary activation through the recognition of antigen A, followed by secondary validation and cytotoxic execution upon detection of antigen B, this approach substantially enhances tumor discrimination.^[^ ^93^ ^]^ Conversely, the IF‐BETTER gate strategy introduces a more advanced mechanism, initially achieving activation through the recognition of antigen A, with subsequent detection of antigen B leading to enhanced activation kinetics and effector function enhancement. This is particularly beneficial in scenarios where antigen A is depleted while antigen B remains present.^[^ ^94^ ^]^ This dynamic modulation of the response optimizes both the intensity of antitumor activity and the persistence of CAR‐T cells within the complex TME. Several designs of CARs based on logic gates have shown significant preclinical efficacy, with some progressing to early‐stage clinical testing. For example, the AND‐gated CAR‐T is currently undergoing evaluation in a Phase I clinical trial (NCT05736731). Nevertheless, the majority of these systems are still in the translational and early clinical research phases.

The logic gate strategy enhances specificity through the recognition of multiple antigens. However, it faces challenges, including the difficulty in identifying suitable antigen pairs due to tumor heterogeneity. Moreover, complex designs may lead to increased manufacturing costs and reduced T‐cell efficacy, which could result in antigen escape or suppression by the TME. Additionally, the requirement for dual antigens in the AND‐gate may diminish sensitivity, whereas the OR‐gate may elevate the risk of off‐target effects. Despite these challenges, this strategy has demonstrated superior efficacy compared to single‐target approaches in solid tumor models. If signal integration is optimized through synthetic biology, this method has the potential to significantly improve clinical outcomes.

In addition to these strategies, suicide gene safety switches represent another important class of logic gates. A prominent example is the inducible caspase‐9 (iC9) system. The iC9 safety switch functions through a chemically induced dimerization (CID) mechanism, centered on the fusion of the iC9 protein with a FKBP12 mutant (F36V). Upon exposure to CID small molecules such as AP1903, iC9 rapidly forms dimers and activates the caspase cascade, thereby triggering CAR‐T cell apoptosis. This mechanism is positioned as an “emergency brake” system, allowing for the rapid elimination of over‐activated CAR‐T cells in cases of severe cytokine release syndrome (CRS ≥ grade 3) or uncontrollable toxicity. Clinical data demonstrate its efficacy in clearing over 90% of CAR‐T cells (within 24 h), along with a significant reduction in levels of inflammatory cytokines such as IL‐6 and IFN‐γ. Compared to traditional suicide genes such as HSV‐TK, iC9 directly induces apoptosis without the involvement of the immune system, offering technical advantages such as rapid action (onset < 6 h) and the absence of immunogenicity risk, thus providing precise and controllable safety assurance for CAR‐T therapy.^[^ ^5^, ^95^ ^]^ NCT04157656 demonstrates a CAR‐T cell clearance rate exceeding 90% following switch activation. The iC9 safety switch has advanced to Phase I clinical trials (e.g., NCT06347068, NCT04650451), where it has shown effective regulation of CAR‐T cell activity and improved clinical safety, exemplifying a successful case of bench‐to‐bedside translation (**Table** **2**).^[^ ^79^, ^84^, ^96^, ^97^, ^98^ ^]^ The iC9 suicide switch functions as a crucial safety mechanism for the effective management of toxicity. However, it presents certain drawbacks, including the potential for incomplete cell clearance, as a residue of less than 10% may lead to persistent CRS. Moreover, optimizing the timing of inducer administration poses challenges, which can delay responses during acute toxicity events. Additionally, the incorporation of iC9 may slightly impact the persistence of CAR‐T cells. While clinical data indicate a clearance rate exceeding 90%, the combination of this system with dose optimization could be essential for high‐risk patients. Nevertheless, further trials are necessary to assess its long‐term immunogenicity.^[^ ^99^ ^]^

**Table 2:** Representative synthetic biology‐based CAR‐T strategies and their progress from bench to bedside.

Mechanism	Category	Representative study	Application model	Translational status	Translational challenge	References
Spatiotemporally controllable CAR	Optogenic control	Dou et al. *Nat Commun*. 2023	Mouse colorectal cancer	Preclinical research	The limited depth of light transmission makes it difficult to precisely control the light source.	[79]
	Sonogenic control	Liu et al. *Cell*. 2025	Human glioblastoma and prostate cancer	Phase I clinical trial (NCT06051695)	The accuracy and safety of heat‐sensitive components are difficult to control.	[84]
Small molecule‐inducible CAR	Dox	Gu et al. *Int J Mol Sci*. 2018	Human CD19^+^ B‐cell leukemia	Preclinical research	The complexity of the regulation system, the heavy burden on the carrier, and the difficulty in pharmacokinetic control.	[96]
	AP20187	Xiao et al. *Cancer Gene Ther*. 2024	Human and mice rhabdomyosarcoma	Preclinical research		[97]
	AP1903	Unpublished	Human advanced tumors expressing PSCA	Phase I clinical trial (NCT02744287)		‐
Logic gate‐engineered CAR	AND gate	Unpublished	Human colorectal cancer	Phase I clinical trial (NCT05736731)	The complexity of antigen selection, the difficulty in regulating signal strength, and the immaturity of manufacturing processes.	‐
	OR gate	Unpublished	Human B‐cell non‐Hodgkin's lymphoma	Phase I/II clinical trial (NCT04007029)		‐
	IF‐THEN gate	Dharani et al. *Mol Ther*. 2024	Human tumor cells expressing mesothelin Human tumor cells expressing mesothelin and FAP Human triple‐negative breast cancer	Preclinical research		[98]
	Inducible caspase‐9‐based system	Unpublished	Human triple‐negative breast cancer	Phase I clinical trial (NCT06347068)	The side effects of activators and the introduction of iCasp9 can affect the persistence and anti‐tumor activity of CAR‐T cells.	‐
		Unpublished	Human HER2 positive advanced solid tumors	Phase I clinical trial (NCT04650451)		‐

### 2.4 AI‐Driven Protein Design for Enhanced CAR‐T Cell Therapy

Traditional CAR‐T cells typically utilize single‐chain variable fragments (scFv) as antigen‐binding domains; however, scFv is characterized by poor stability, susceptibility to aggregation, and degradation, which ultimately limits its efficacy. The rapid advancement of artificial intelligence presents an optimized solution to these challenges. Nobel Prize winner David Baker has further developed RFdiffusion, allowing for the flexible design of adhesive components by refining input structural models through iterative noise application and denoising.^[^ ^44^ ^]^ Additionally, pyrazole affinity ligands can be improved based on designs generated by alternative methodologies or can be initiated entirely de novo from random noise distributions, eliminating the necessity for subsequent experimental optimization.^[^ ^44^, ^100^, ^101^ ^]^

RFdiffusion is intricately linked to Rosetta, as both are pivotal tools in the realm of protein design. RFdiffusion, a protein structure generation tool, is developed using technologies related to Rosetta. Rosetta primarily employs knowledge of protein energy landscapes to derive amino acid sequences by optimizing protein fragments, concentrating on optimization that begins with known structural fragments. In contrast, RFdiffusion integrates diffusion models and deep neural networks, demonstrating a superior capacity for generating protein structures that fulfill specific target functions. Together, these two tools can enhance protein research, offering more comprehensive solutions for protein structure prediction and design.

Furthermore, researchers have utilized artificial intelligence‐driven protein design software, such as Rosetta and AlphaFold2, to create high‐affinity protein binders (DNDB) *de novo*, providing alternatives to traditional scFv for antigen recognition in CAR‐T cells.^[^ ^47^ ^]^ Experimental findings indicate that DNDB‐CAR‐T cells exhibit superior anti‐tumor activity compared to conventional scFv‐CAR‐T cells, demonstrating enhanced proliferative capacity, increased secretion of cytotoxic factors, and improved resistance to exhaustion in both in vitro and in vivo settings. Additionally, DNDB‐CAR‐T cells show greater stability in surface expression and enhanced resistance to degradation within the TME, significantly improving their persistence and therapeutic efficacy.^[^ ^47^ ^]^

However, several limitations associated with Rosetta software merit consideration, including potential inaccuracies in predictions, substantial computational resource demands, and limited disclosure of certain technical parameters, which could affect practical implementation and research reproducibility.^[^ ^100^ ^]^ Despite these challenges, Rosetta software remains a powerful computational platform for advancing personalized therapeutic strategies in the treatment of solid tumors.^[^ ^44^ ^]^ Future research should concentrate on optimizing prediction accuracy, enhancing computational efficiency, and improving technical transparency to fully realize its potential in translational applications. While RFdiffusion exhibits strong generative capabilities in *de novo* protein design, its deep learning model's “black box” nature—characterized by the opacity of its decision‐making processes—may limit mechanistic insights into the generated structures. This limitation could hinder targeted optimizations and the identification of potential biases.^[^ ^102^ ^]^ Furthermore, the challenges associated with validating AI‐generated targets include the lengthy and costly nature of experimental confirmation. In vitro and in vivo assessments of protein structures require extensive biochemical assays and evaluations using animal models, which present a significant challenge, especially for resource‐constrained laboratories^[^ ^103^ ^]^ Despite these limitations, integrating interpretable AI techniques or hybrid physical modeling may enhance the reliability of this approach in CAR‐T cell optimization.

### 2.5 Enhanced CAR‐T: Cytokine Armor Based on Synthetic Biology

#### 2.5.1 Modulation of Tumor Immune Microenvironment to Enhance CAR‐T Cell Therapy Efficacy

The TME in solid tumors poses significant challenges to the efficacy of CAR‐T cell therapy through various mechanisms.^[^ ^55^ ^]^ First, the cytokine networks present within the TME can substantially impair the functionality of CAR‐T cells, thereby diminishing their tumoricidal capacity.^[^ ^104^ ^]^ Second, the presence of immunosuppressive cells and the biochemical milieu within the TME may lead to CAR‐T cell exhaustion.^[^ ^105^, ^106^ ^]^ Furthermore, tumor cells utilize sophisticated immune evasion strategies, including the secretion of immunosuppressive factors and the recruitment of inhibitory immune cells.^[^ ^107^, ^108^ ^]^ Collectively, these mechanisms create an immunosuppressive environment that undermines anti‐tumor immune responses, ultimately compromising therapeutic outcomes. Therefore, a comprehensive understanding and strategic modulation of the tumor immune microenvironment are essential for enhancing the efficacy of CAR‐T cell therapy.

**Figure 4:**
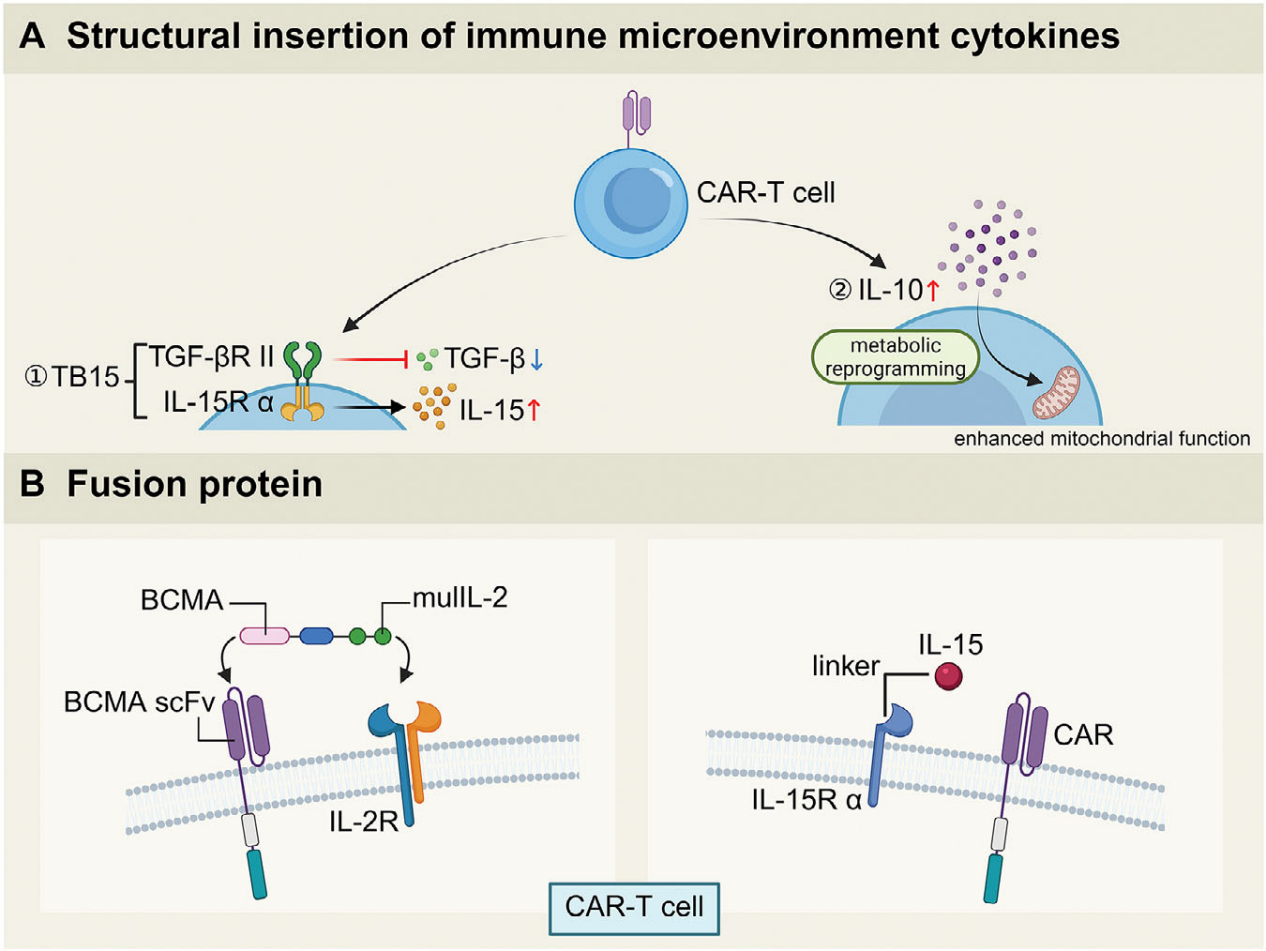
The engineering of CAR‐T cells with immune microenvironment‐responsive cytokine systems. A) Structural insertion of immune microenvironment cytokines: CAR‐T cells are engineered to incorporate receptors for immune cytokines, specifically TGF‐βR II, and IL‐10, in order to enhance their functionality and survival within the TME. B) Fusion protein design: CAR‐T cells are equipped with fusion proteins that connect tumor‐targeting elements, such as BCMA scFv, to immune modulatory cytokine receptors, including IL‐15R α and IL‐2R (Created by BioRender).

Deng and colleagues recently developed a novel immune cell receptor (ICR) structure, designated TB15, by fusing the extracellular domain of TGF‐β receptor II (TGF‐βRII) with the intracellular domain of IL‐15 receptor α (IL‐15Rα).^[^ ^105^ ^]^ TB15‐modified CAR‐T cells exhibited remarkable anti‐tumor activity in environments characterized by high levels of TGF‐β, effectively inhibiting TGF‐β signaling while simultaneously activating IL‐15‐mediated stimulation. This dual action enhances the persistence and functionality of CAR‐T cells. In murine models, TB15‐modified CAR‐T cells significantly suppressed tumor growth and extended survival. The innovative design of the TB15 ICR improves CAR‐T cell function within immunosuppressive TMEs through two mechanisms: reversing TGF‐β signaling and activating IL‐15 signaling. Although TB15‐modified CAR‐T cells show promising anti‐tumor efficacy and persistence in solid TME, further research is necessary to elucidate the precise molecular mechanisms and assess the long‐term safety profile of the TB15 ICR (**Figure** **4**Ai).^[^ ^105^ ^]^

Prior to this advancement, IL‐15, a cytokine recognized for promoting T cell survival and function, had been integrated into CAR‐T cell therapy to enhance anti‐tumor activity.^[^ ^109^ ^]^ Professor Andras Heczey and colleagues were the first to validate the efficacy of IL‐15‐enhanced CAR‐T cells in human clinical trials. In a study involving 24 patients—12 receiving GPC3 CAR‐T cells and 12 receiving 15.CAR treatment—15. CAR T cells demonstrated significant in vivo expansion, achieving a 66% disease control rate and a 33% objective response rate, thus substantially outperforming conventional CAR‐T cells.^[^ ^5^ ^]^ However, the use of 15. CAR T cell therapy has been linked to a heightened incidence of CRS, which can be effectively managed through the application of IL‐1/IL‐6 blockade or the implementation of inducible caspase 9 safety switches. Furthermore, single‐cell RNA sequencing analysis has demonstrated that IL‐15‐modified CAR‐T cells possess enhanced effector functions and improved persistence within the TME. This enhancement correlates with the activation of FOS/JUN family members and the engagement of type I interferon signaling pathways. These findings not only affirm the efficacy of IL‐15 co‐expression strategies but also offer novel insights into the in vivo evolution of CAR‐T cells.

Contrary to conventional understanding, which typically associates IL‐10 overexpression in the tumor immune microenvironment with immune evasion and immunosuppression,^[^ ^110^ ^]^ recent studies conducted by Tang Li and colleagues have revealed unexpected findings. Their research, employing comprehensive evaluations through multiple murine models and advanced technologies such as single‐cell RNA sequencing and metabolomics, demonstrates that moderate IL‐10 overexpression in CAR‐T cells can enhance mitochondrial function via metabolic reprogramming. This process effectively overcomes T cell exhaustion in solid tumors, significantly improving anti‐tumor efficacy (Figure 4Aii).^[^ ^111^ ^]^ This metabolic reprogramming strategy provides novel mechanistic insights into the enhancement of CAR‐T cells. While potential differences between murine models and human TMEs may impact clinical translation, the broad applicability of metabolic reprogramming strategies presents a promising new direction for enhancing CAR‐T cell efficacy in solid tumors.^[^ ^112^ ^]^ Moreover, recent studies indicate that huCART19‐IL18 therapy demonstrates efficacy in lymphoma patients who have previously failed anti‐CD19 CAR T cell therapy. Its safety profile is comparable to that of other CAR T cell treatments, thereby offering a new treatment option for these patients.^[^ ^113^ ^]^

#### 2.5.2 Boosting CAR‐T Cell Efficacy with Cytokine‐Based Fusion Proteins

Clinical trials have consistently shown that the uncontrolled, constitutive expression of cytokines to enhance the efficacy of CAR‐T cells often results in severe CRS and CAR‐T cell exhaustion.^[^ ^114^ ^]^ This phenomenon primarily arises from activated T cells rapidly producing large quantities of cytokines, such as IL‐2 and IL‐15, which significantly intensify the immune response, thereby triggering severe CRS.^[^ ^115^ ^]^ Notably, IL‐2 has multifaceted roles within the immune system; it is not only a critical factor for T cell proliferation but also a key regulator of immune responses and the maintenance of immune homeostasis.^[^ ^116^ ^]^ Consequently, the targeted modification of functional domains based on the specific functions of cytokines has emerged as a vital strategy in the design of CAR‐enhancers (CAR‐E), providing a more precise approach to balance therapeutic efficacy with safety (Figure 4B).

The study conducted by Mohammad Rashidian et al. demonstrated that BCMA‐mutIL‐2 CAR‐E can gradually and persistently enhance the efficacy of CAR‐T cells, supporting multiple courses of monotherapy for up to 97 days.^[^ ^117^ ^]^ While IL‐15 shares several functional similarities with IL‐2, it is particularly crucial for the generation of durable, highly active memory CD8^+^ T cells.^[^ ^118^, ^119^ ^]^ IL‐15 forms a stable complex by binding to IL‐15Rα, which subsequently interacts with the β and γ chain receptors to activate downstream signaling pathways.^[^ ^120^ ^]^ This mechanism is analogous to that of IL‐2; however, IL‐15 primarily supports the survival and proliferation of memory T cells and NK cells without significantly promoting the expansion of regulatory T cells.^[^ ^121^ ^]^ In response to these findings, Novartis has developed NIZ985, an IL‐15/IL‐15Rα fusion protein,^[^ ^122^, ^123^, ^124^ ^]^ which is utilized in combination with immune checkpoint inhibitors to enhance antitumor efficacy in solid tumors. Furthermore, Genentech (Roche) has demonstrated the preclinical efficacy of its IL‐15/IL‐15Rα‐Fc fusion protein, with clinical outcomes currently under evaluation. Although the application of IL‐15/IL‐15R trimeric fusion proteins in induced natural killer (iNK) cells has been reported,^[^ ^125^ ^]^ their use in CAR‐T cells remains limited. Nevertheless, this approach continues to represent a viable strategy for CAR‐E design.

### 2.6 Advances in Experimental Models for Solid Tumor CAR‐T Cell Therapies

While ongoing advancements in CAR‐T cell engineering are crucial, the development of experimental models that accurately replicate the complex TME is equally essential for advancing solid tumor CAR‐T cell therapies. Such models serve to bridge the gap between preclinical research and clinical efficacy, facilitating mechanistic insights and therapeutic optimization.

Traditional 2D cell culture systems are inadequate in capturing the intricate interactions between tumor cells and their microenvironment. In contrast, 3D culture models, such as tumor spheroids and patient‐derived organoids, offer more physiologically relevant platforms for studying tumor growth dynamics and cellular interactions. A seminal study by Donald M. O'Rourke and colleagues demonstrated the utility of glioblastoma organoids (GBOs) as real‐time surrogates for evaluating the responses to clinical CAR‐T cell therapy. Their findings indicated that CAR‐T cell treatment led to target antigen reduction and tumor cell lysis in GBOs, with the extent of lysis correlating with CAR‐T cell engraftment levels in patient cerebrospinal fluid (CSF).^[^ ^126^ ^]^ Furthermore, the cytokine release patterns observed in GBOs mirrored those found in patient CSF samples. These results establish GBOs as a valuable platform for the real‐time assessment of CAR‐T cell bioactivity, thereby offering new avenues for personalized medicine.^[^ ^127^ ^]^ This approach not only aids in the optimization of clinical trial design but also provides critical insights for the development of future therapeutic strategies.

The limited understanding of CAR‐T cell distribution and biological behavior within solid tumors poses a significant barrier to effective therapeutic monitoring in solid tumor immunotherapy. Advanced imaging technologies have emerged as essential tools for tracking CAR‐T cell migration, distribution, and tumor targeting in vivo, offering valuable insights into the microenvironmental influences on CAR‐T cell survival and efficacy. Positron Emission Tomography (PET) facilitates the tracking of CAR‐T cell distribution and metabolic activity through radionuclide labeling, thereby enhancing the assessment of tumor targeting and therapeutic response.^[^ ^128^ ^]^ Magnetic Resonance Imaging (MRI), which utilizes magnetic nanoparticle labeling, provides high‐resolution visualization of CAR‐T cell distribution and migration patterns, allowing for precise evaluation of tumor infiltration. Additionally, optical imaging techniques that employ fluorescent dyes or bioluminescent proteins enable real‐time, dynamic monitoring of CAR‐T cell migration, distribution, and tumor targeting, yielding critical insights into interactions within the TME.

A significant advancement was achieved by Mohammad Rashidian and colleagues, who developed and synthesized a CD19 antigen‐based PET probe. This probe, labeled with 89Zr‐DFO‐PEG20, demonstrated specific binding and imaging of CAR‐T cells, representing the first validation of antigen‐based CAR‐PET imaging in immunocompetent models.^[^ ^129^ ^]^ However, challenges persist in clinical translation, including the complexity of probe synthesis, the limitations of animal models, and the accuracy of predictive models. Future directions should focus on optimizing the synthesis and labeling processes of the probe for large‐scale clinical applications, as well as developing more accessible imaging modalities, such as ultrasound probes, to enhance clinical feasibility.

### 2.7 Fabrication of Universal CAR‐T

Conventional autologous CAR‐T cell therapy involves the extraction of T cells from patients, followed by genetic engineering and reinfusion. This process is complex and time‐consuming, with limitations arising from patient variability and cell quality.^[^ ^130^ ^]^ In contrast, ‘off‐the‐shelf’ universal CAR‐T cell therapy utilizes induced pluripotent stem cells (iPSCs) as a cell source, facilitating large‐scale in vitro production. This innovation significantly streamlines the manufacturing process and reduces costs.^[^ ^131^, ^132^ ^]^ Furthermore, it provides standardized products that are suitable for broader patient populations, thereby enhancing treatment accessibility.^[^ ^133^ ^]^

A groundbreaking study by George Q. Daley and colleagues employed a stroma‐free iPSC‐T cell differentiation platform for small molecule screening, identifying G9a/GLP as an epigenetic regulator of T cell fate. By chemically inhibiting G9a/GLP, the researchers successfully generated functionally mature CAR‐T cells from human iPSCs, presenting a novel strategy for off‐the‐shelf immunotherapy.^[^ ^134^ ^]^ While these CAR iPSC‐T cells demonstrated enhanced anti‐tumor activity both in vitro and in vivo—particularly in tumor rechallenge models where they exhibited persistent tumor clearance and therapeutic potential—challenges remain. iPSC‐derived T cells often display immature phenotypes with functional limitations compared to peripheral blood‐derived mature T cells.^[^ ^135^ ^]^ Although the chemical inhibition of G9a/GLP has successfully generated functionally mature CAR‐T cells, further optimization of differentiation conditions is necessary to ensure cellular functionality and stability.

The allogeneic nature of off‐the‐shelf CAR‐T cells derived from iPSCs also presents challenges, including the potential for immune rejection in recipients. Future research must address critical issues such as reducing immunogenicity through gene editing technologies and minimizing epigenetic memory in iPSCs to advance this promising therapeutic approach.

Furthermore, to address the critical challenge of immune rejection in allogeneic cell therapies, researchers have drawn inspiration from viral immune evasion mechanisms.^[^ ^136^, ^137^ ^]^ Notably, viruses such as HIV, EBV, and CMV, which have evolved sophisticated strategies to circumvent immune surveillance, provide valuable templates for bioengineering.^[^ ^130^, ^138^ ^]^ A groundbreaking study by Prof. Michel's group demonstrated the innovative repurposing of HIV‐1's Nef protein—a potent immune evasion factor—to engineer allogeneic CAR‐T cells. By downregulating surface MHC‐I expression, these modified CAR‐T cells effectively evade host T cell‐mediated rejection, thereby achieving prolonged persistence and enhanced antitumor efficacy.^[^ ^139^ ^]^ This “pathogen‐inspired engineering” paradigm overcomes the historical limitations of allogeneic CAR‐T therapies constrained by immunogenicity, offering a transformative approach for developing universal “off‐the‐shelf” cellular products.

The study not only validates the translational potential of repurposing pathogen‐derived evasion tactics in synthetic immunology but also establishes a novel framework for rebalancing immune cell surface molecules to mitigate host‐versus‐graft responses. Further integration with complementary technologies, such as TCR gene editing, could accelerate the clinical translation of allogeneic CAR‐T therapies. However, systematic evaluation of safety concerns—including increased infection susceptibility due to MHC‐I suppression, potential oncogenic risks associated with viral protein expression, and residual graft‐versus‐host disease (GVHD) risks from incomplete TCR silencing—remains imperative to advance this strategy toward clinical applicability.

## 3 Advancing Clinical Protocols for CAR‐T Cell Therapy in Solid Tumor Treatment

### 3.1 Surgical‐Assisted Delivery Strategies for CAR‐T Therapy in Solid Tumors

**Figure 5:**
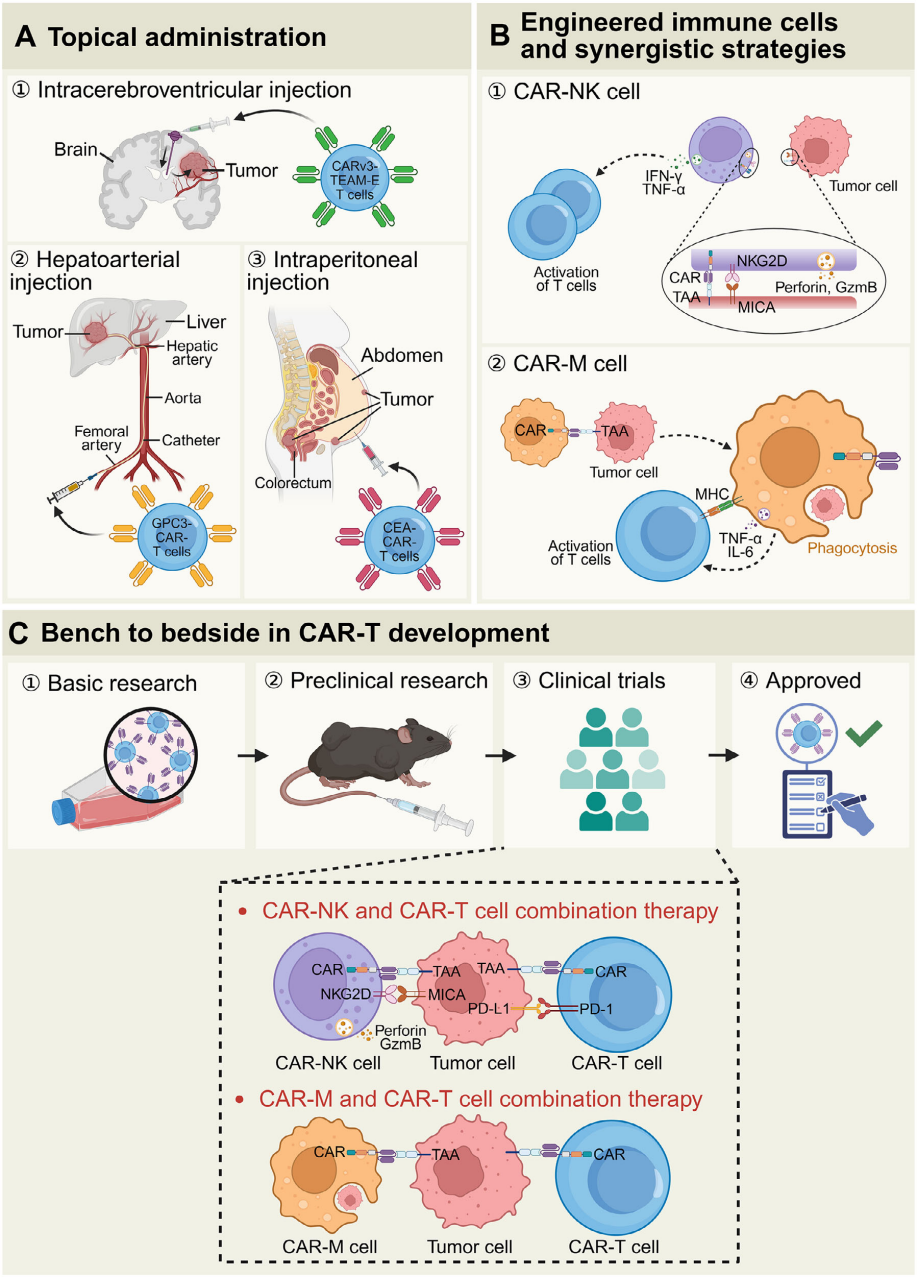
An overview of localized administration, engineered immune cell strategies, and the translational roadmap in CAR‐T therapy. A) The topical administration methods for CAR‐T cell therapies targeting specific tumors. CAR‐T cells, engineered to recognize tumor antigens (e.g., EGFR for brain tumors, GPC3 for liver tumors, and CEA for colorectal tumors), are delivered through various routes, including intracerebroventricular, hepatoarterial, and intraperitoneal injections. B) The types of engineered immune cells and their synergistic strategies. ① CAR‐NK cells induce tumor lysis through CAR, NKG2D, and the release of cytotoxic granules, while also enhancing T‐cell activation via cytokine secretion (e.g., IFN‐γ, TNF‐α). ② CAR‐M cells facilitate tumor phagocytosis and antigen presentation, reshaping the TME through pro‐inflammatory cytokines (e.g., TNF‐α, IL‐6). C) The CAR‐T translational pipeline encompasses the progression from basic and preclinical research to clinical trials and eventual approval. The bottom diagram depicts the combination of CAR‐NK or CAR‐M cells with CAR‐T cells, which can enhance their efficacy (Created by BioRender).

A groundbreaking study conducted by Boston Marcela V. Maus has introduced a novel CAR T cell therapy, termed CARv3‐TEAM‐E, which targets both the EGFRvIII mutation and wild‐type EGFR protein. This innovative therapy also secretes T cell‐engaging antibody molecules (TEAM) to address the limitations associated with single‐antigen targeting. Notably, despite previous unsuccessful attempts to target EGFRvIII,^[^ ^45^ ^]^ this study utilized a single‐center, open‐label design, administering a single intracerebroventricular infusion of 1 × 10^7^ CARv3‐TEAM‐E T cells (**Figure** **5**A). Clinical trial efficacy data indicated that no patients experienced grade 3 or higher adverse events or dose‐limiting toxicities. Radiographic assessments demonstrated rapid tumor regression within days following the infusion; however, two patients exhibited only transient responses, while one patient showed durable remission. Liquid biopsy analyses revealed significant reductions in both EGFRvIII and EGFR copy numbers in cerebrospinal fluid and peripheral blood post‐treatment. The study's innovative approach transcends the CAR‐T cell design, as the intracerebroventricular administration route signifies a notable advancement. This delivery method not only provides direct access to central nervous system tumors but also enhances the local concentration of the drug at the tumor site while minimizing systemic toxicity.

In the realm of localized CAR‐T cell therapy for solid tumors, notable advancements have been achieved not only in glioblastoma but also in hepatocellular carcinoma (HCC) and metastatic gastrointestinal tumors. An early‐phase clinical trial (NCT02932956) assessed the safety and preliminary efficacy of hepatic artery infusion of GPC3‐CAR‐T cells in patients with HCC, revealing tumor regression in several cases alongside manageable toxicity profiles.^[^ ^5^ ^]^ Furthermore, exploratory clinical studies have been undertaken for metastatic gastrointestinal tumors. A phase I trial (NCT02349724) examined the intraperitoneal administration of CEA‐CAR‐T cells in colorectal cancer patients, demonstrating tumor reduction in certain instances with acceptable toxicity.^[^ ^140^ ^]^ Similarly, an early‐phase clinical trial (NCT02414269) evaluated the intraperitoneal delivery of MSLN‐CAR‐T cells in ovarian cancer patients, with preliminary findings indicating tumor regression in some subjects and manageable adverse effects.^[^ ^141^ ^]^ However, in contrast to glioblastoma and hepatic parenchyma, the intricate peritoneal microenvironment poses distinct challenges, including the presence of immunosuppressive cells such as Tregs and MDSCs ^[^ ^142^, ^143^ ^]^, as well as inhibitory molecules like PD‐L1^[^ ^144^ ^]^, which may impede the efficacy of CAR‐T cells. Future research should prioritize multi‐target designs, combination therapies, and technical optimizations to enhance the therapeutic efficacy and safety profile of intraperitoneal CAR‐T cell therapy for metastatic gastrointestinal and ovarian cancers (Figure 5A).

### 3.2 Innovations in Internal Medicine

#### 3.2.1 Emerging Strategies in Combined Treatment Exploration

Research has demonstrated that CAR‐T cells are frequently influenced by inhibitory factors secreted by tumor cells (**Table** **3**). Immune checkpoint inhibitors, such as PD‐1/PD‐L1 inhibitors, can mitigate this inhibition, thereby enhancing the functionality and persistence of CAR‐T cells.^[^ ^21^ ^]^ Recent clinical trials have indicated that the combination of CAR‐T cell therapy with CTLA‐4 inhibitors exhibits synergistic effects in suppressing the progression of non‐small cell lung cancer (NCT03430080), resulting in improved progression‐free survival (PFS) and overall survival (OS). Additionally, ongoing evaluations are investigating the efficacy of CAR‐T cell therapy in conjunction with PD‐1 inhibitors for patients with advanced melanoma (NCT03356730). The findings from these studies suggest that while combination therapies enhance therapeutic efficacy, they also elevate the risk of immune‐related adverse events (irAEs).^[^ ^145^ ^]^ Overall, the integration of CAR‐T cell therapy with immune checkpoint inhibitors presents promising potential for the treatment of solid tumors, and research in this area continues to progress. Future efforts will necessitate further clinical trials to validate long‐term efficacy and safety, as well as to explore optimal treatment combinations and personalized strategies.

**Table 3:** Clinical trials of CAR‐T in combination with PD‐1/PD‐L1 inhibitor or with CAR‐NK/CAR‐M therapy.

Trial ID	Cell types	CAR targets	Combination Strategy	Cancer Type	Phase	Status	Sponsor / Institution
NCT03874897	CAR‐T	Claudiwn18.2	PD‐1 inhibitor	Claudin18.2‐positive digestive system cancers (e.g. FC, GEJ, PC)	I	Completed	Peking University
NCT04581473	CAR‐T	Claudin18.2	PD‐1 inhibitor	Claudin18.2‐positive digestive system cancers (e.g. FC, GEJ, PC)	II	Active	Peking University
NCT03615313	CAR‐T	Mesothelin	PD‐1 inhibitor	Advanced solid tumors	I/II	Unknown	Shanghai Cell Therapy Research Institute
NCT04134325	CAR‐T	CD30	PD‐1 inhibitor (Nivolumab or Pembrolizumab)	Relapsed/Refractory classical Hodgkin lymphoma	I	Active	UNC Lineberger Comprehensive Cancer Center
NCT04850560	CAR‐T	CD28	PD‐1 inhibitor	Relapse/Refractory B‐cell lymphoma	I	Unknown	Zhejiang University
NCT05631899	CAR‐T	KRAS	PD‐1 inhibitor	Local advanced/Metastatic solid tumors	I	Recruiting	Chinese PLA General Hospital
NCT05631886	CAR‐T	TP53	PD‐1 inhibitor	Local advanced/Metastatic solid tumors or Relapsed/Refractory lymphomas	I	Recruiting	Chinese PLA General Hospital
NCT03233854	CAR‐T	CD19/CD22	CAR‐NK	B‐cell acute lymphoblastic leukemia	I	Active	Crystal Mackall
NCT03383978	CAR‐NK	HER‐2	PD‐1 inhibitor (Ezabenlimab)	Recurrent HER2‐positive glioblastoma	I	Active	Johann Wolfgang Goethe University Hospital
NCT04660929	CAR‐M	HER‐2	PD‐1 inhibitor (Pembrolizumab)	HER2 overexpressing solid tumors	I	Active	Carisma Therapeutics

#### 3.2.2 Leveraging Synergistic CAR‐NK and CAR‐T Cell Strategies for Cancer Immunotherapy

Natural killer (NK) cells are activated through a dual mechanism involving ‘missing‐self’ recognition, which identifies downregulated MHC‐I, and ‘induced‐self’ recognition, which detects stress ligands such as NKG2D. This activation is independent of HLA, thus preventing graft‐versus‐host disease (GvHD) (Figure 5B). A Phase I trial conducted by Qian et al. in 2025 (NCT05472558) revealed that allogeneic CAR‐NK cell therapy administered to 28 patients with relapsed/refractory large B‐cell lymphoma (R/R LBCL) resulted in no cases of GvHD, contrasting with a 22% incidence rate of GvHD observed in contemporaneous allogeneic CAR‐T cell therapies.^[^ ^146^ ^]^ The off‐the‐shelf availability of CAR‐NK cells enhances accessibility compared to CAR‐T cells. Currently, both umbilical cord blood (UCB) and iPSC‐derived CAR‐NK cells are undergoing standardized production.^[^ ^147^ ^]^ For instance, For example, UCB‐CAR‐NK cells targeting CLDN18.2 in a gastric cancer model demonstrated a 14‐day survival advantage and a 70% reduction in tumor volume following allogeneic infusion, without provoking immune rejection (NCT06464965).

The allogeneic compatibility (absence of GvHD), low toxicity (negligible cytokine release syndrome, CRS, and immune effector cell‐associated neurotoxicity syndrome, ICANS), and off‐the‐shelf potential of CAR‐NK cells position them as a safer and more accessible alternative to CAR‐T cells. By 2025, six CAR‐NK therapies are expected to receive global approval (three for hematological malignancies and three for solid tumors), with a projected market share of 30% within the cell therapy landscape by 2030. Advances in iPSC technology and genetic circuitry suggest that CAR‐NK cells are likely to evolve from being considered ‘complementary therapies’ to first‐line treatments for solid tumors, especially for patients with CAR‐T‐resistant or intolerant diseases. Despite these advantages, the persistence of NK cells has been reported as suboptimal, with a cellular half‐life of less than seven days. Strategies to enhance NK cell survival include IL‐15 transgene expression and metabolic reprogramming.^[^ ^148^ ^]^ Furthermore, the limited infiltration of NK cells into solid tumors has led to the development of CXCR4‐modified CAR‐NK cells, which have demonstrated a two‐fold increase in infiltration rate during a Phase I trial for glioblastoma (NCT06234567). However, challenges persist in the engineered production and *ex vivo* expansion of these cells. As of 2025, the FDA has approved the first iPSC‐NK production line by Allogene Company, aimed at scaling up the manufacturing of iPSC‐CAR‐NK cells.

Leveraging the mechanisms of action of CAR‐NK cells, both natural cytotoxicity (perforin/granzyme) and antibody‐dependent cell‐mediated cytotoxicity (ADCC) can effectively eliminate circulating tumor cells (CTCs), while CAR‐T cells specifically target the primary lesions. This synergistic strategy has demonstrated that the CAR‐NK‐mediated ‘clearance of metastatic sentinels,’ combined with the CAR‐T‐mediated ‘destruction of primary fortresses,’ can systemically disrupt the ‘metastasis‐recurrence’ cycle in melanoma, thereby doubling the complete remission rate (Table 3).^[^ ^149^ ^]^ The core innovation lies in the precise division of labor that targets the ‘dynamic life cycle’ of the tumor, rather than relying on simple superposition. With advancements in iPSC‐CAR‐NK cells and memory CAR‐T cells, this model is well‐positioned to become the ‘standard combination’ for treating solid tumors, such as advanced melanoma and pancreatic cancer, particularly for patients exhibiting resistance to CAR‐T monotherapy or those at high risk of metastasis.

#### 3.2.3 TME Orchestration: CAR‐M and CAR‐T Collaboration for Anti‐Tumor Immunity

Macrophages play a crucial role in the body's immune response, not only through their potent phagocytic ability to eliminate pathogens and aberrant cells but also through their involvement in antigen presentation and immune modulation.^[^ ^150^ ^]^ CAR‐M cells, engineered to express a CAR on their surface, are capable of specifically recognizing antigens on tumor cells. Upon recognition, CAR‐M cells initiate phagocytosis, engulfing and degrading the tumor cells. For instance, CT‐0508, a CAR‐M therapy targeting HER2‐positive solid tumors, expresses an anti‐HER2 CAR that precisely recognizes and efficiently phagocytoses HER2‐overexpressing tumor cells, thereby directly reducing the tumor cell population.^[^ ^151^ ^]^ In addition to phagocytosis, CAR‐M cells can secrete cytotoxic substances such as perforin and granzymes, which induce tumor cell apoptosis. Furthermore, activated CAR‐M cells can trigger apoptotic pathways in tumor cells through death receptor signaling, including the Fas/FasL and TNF‐α/TNFR1 pathways.^[^ ^152^ ^]^ In vitro experiments have demonstrated that CAR‐M cells induce changes in the expression of apoptosis‐related proteins in tumor cells, confirming their ability to trigger tumor cell apoptosis. In various murine models of solid tumors, CAR‐M cell therapy has shown promising therapeutic efficacy. A second‐generation CAR‐iMAC, constructed by Zhang *et al.* at Zhejiang University, resulted in complete tumor elimination in six out of eight treated mice in an HCC model and significantly prolonged survival in a glioblastoma‐bearing mouse model.^[^ ^153^ ^]^

Moreover, the TME is frequently characterized by immunosuppressive properties, which hinder the immune system's capacity to target tumor cells.^[^ ^154^ ^]^ CAR‐M cells can secrete various pro‐inflammatory cytokines, including TNF‐α and IL‐6, which play a crucial role in activating effector T cells, such as CD8^+^ T cells. This activation promotes their infiltration into the tumor site and enhances their cytotoxic capabilities against tumor cells.^[^ ^155^ ^]^ Concurrently, CAR‐M cells can reduce the proportion of immunosuppressive cells, such as Tregs and M2 macrophages, thereby disrupting the immunosuppressive state of the TME.^[^ ^156^, ^157^, ^158^ ^]^ In studies focused on HER2‐positive solid tumors, CAR‐M cell therapy resulted in a significant increase in the number of CD8^+^ T cells and a marked decrease in the proportion of Tregs within the TME, effectively shifting the immune landscape toward an anti‐tumor profile.^[^ ^159^ ^]^ The research team led by Weihong Tan employed an mRNA‐LNP system to facilitate the in situ construction of CAR‐M cells in vivo, leading to robust activation of the adaptive immune system in murine models of solid tumors and demonstrating significant synergistic effects with PD‐1/PD‐L1 immune checkpoint blockade therapy.^[^ ^160^ ^]^

A significant advantage of CAR‐M cells is their intrinsic ability to present antigens, making them a natural partner for CAR‐T cell combination therapy. Following the phagocytosis of tumor cells, CAR‐M cells process the tumor antigens and present them on MHC molecules to T cells, thereby activating T cell immune responses. Furthermore, CAR‐M cells can recruit dendritic cells (DCs), which enhances antigen presentation and amplifies the overall anti‐tumor immune response.^[^ ^161^, ^162^ ^]^ For example, in several mouse tumor models, CAR‐M cell therapy resulted in an increased number of DCs in the tumor‐draining lymph nodes, accompanied by enhanced DC maturation and antigen‐presenting capability, thus promoting T cell activation and proliferation.^[^ ^163^ ^]^ Studies have shown that the combination of CAR‐M and CAR‐T cell therapy extended the median survival of mice by 30% in a glioblastoma model.^[^ ^164^ ^]^ The combined therapy group demonstrated a three‐fold increase in CD8^+^ T cell infiltration, a decrease in the proportion of M2 macrophages from 65% to 22%, and an 80% reduction in tumor volume within the TME.

While the combination of CAR‐M and CAR‐T cells represents a mechanistically complementary and closed‐loop strategy for TME remodeling and efficient tumor cell killing, several challenges persist in the treatment of solid tumors. First, functional interference between the two cell types may occur. The synergy of IL‐6 can trigger severe CRS, with serum IL‐6 levels in the combined therapy group being five times higher than those in the CAR‐T monotherapy group, necessitating the prophylactic administration of tocilizumab.^[^ ^165^ ^]^ Furthermore, competition for antigens or survival space may arise, requiring careful control of the CAR‐M to CAR‐T ratio.^[^ ^166^ ^]^ Second, the heterogeneity of solid tumors may lead to the downregulation of shared antigens, rendering dual therapy ineffective, while the metabolic microenvironment may inhibit CAR‐T cell activity. The most significant translational bottleneck lies in the manufacturing costs associated with these therapies. Standardizing the proportions, sequences, and dosages of co‐infused cell types presents considerable challenges. For example, reports suggest that a combination of 1 × 10^8^ CAR‐M cells and 5 × 10^8^ CAR‐T cells yields optimal efficacy.^[^ ^166^ ^]^ Although a universal strategy could potentially reduce costs by 60%, it carries the risk of immune rejection, necessitating further CRISPR knockout of HLA‐I to mitigate this issue.^[^ ^167^ ^]^

#### 3.2.4 CAR‐Ts and Beyond: Exploring Synergistic Engineered Immune Cells

CAR‐γδ T cells provide the advantage of MHC‐independent recognition, rendering them effective against immune‐desert tumors such as pancreatic cancer. CAR‐γδ T cells targeting GD2 have demonstrated a 60% tumor regression rate in neuroblastoma models.^[^ ^168^ ^]^ In contrast, CAR‐Treg cells can mitigate excessive inflammation in the TME, thereby reducing the risk of CRS. These cells have been shown to improve immune homeostasis by modulating the TGF‐β pathway in murine models.^[^ ^169^ ^]^ Additionally, natural killer T (NKT) cells recognize glycolipid antigens through the CD1d molecule. Upon activation, they secrete substantial quantities of IFN‐γ and IL‐13, exhibiting both Th1 (anti‐tumor) and Th2 (immune‐modulatory) properties. CAR‐NKT cells can enhance the targeting of CD1d‐positive tumors, such as hepatocellular carcinoma and lymphoma, while also reversing immunosuppression by modulating M2 macrophages and Tregs within the TME. Off‐the‐shelf iPSC‐CAR‐NKT cells targeting GPC3, when combined with doxorubicin in a murine model of HCC, resulted in a 90% reduction in tumor volume.^[^ ^170^ ^]^

DCs, known as the most potent antigen‐presenting cells, can be engineered into CAR‐DCs to specifically target tumor antigens such as MUC1, thereby enhancing their accumulation at the tumor site. Following the uptake of antigens, CAR‐DCs migrate to lymph nodes, where they activate both CD8^+^ T cells and CD4^+^ T cells. When combined with CAR‐T cells, CAR‐DCs can significantly improve the priming efficiency and memory formation of CAR‐T cells. In a synovial sarcoma model, the combination of CAR‐DCs targeting NY‐ESO‐1 with CAR‐T cells resulted in an increase in the complete remission rate from 25% with CAR‐T monotherapy to 60%;^[^ ^171^, ^172^ ^]^ similarly, in an NSCLC model, CAR‐DCs targeting WT1 combined with CAR‐T cells targeting MUC1 improved the 1‐year recurrence‐free survival rate from 30% to 70%.^[^ ^173^, ^174^ ^]^

## 4 Conclusion and Prospects

Despite significant advancements in CAR‐T therapy, several critical limitations impede its broader application, particularly in solid tumors. 1) Limited tumor infiltration and persistence. CAR‐T cells often demonstrate inadequate penetration into solid tumors due to dense ECM networks and the presence of immunosuppressive cytokines, with only 1–2% of infused cells reaching the tumor core in preclinical models.^[^ ^175^ ^]^ For instance, pancreatic ductal adenocarcinoma exhibits less than 0.5% CAR‐T infiltration, a phenomenon attributed to physical exclusion mediated by fibrotic stroma.^[^ ^176^ ^]^ 2) Antigen heterogeneity and immune escape. Tumor cells frequently downregulate target antigens or transition to antigen‐negative subclones, resulting in treatment failure in 30–40% of patients.^[^ ^177^ ^]^ Single‐cell sequencing has revealed that only 62% of glioblastoma cells express EGFRvIII, the target of CAR‐T therapy, contributing to incomplete responses.^[^ ^178^ ^]^ 3) Safety concerns. Severe CRS (≥ grade 3) occurs in 15–30% of CAR‐T recipients, driven by excessive release of IL‐6 and IFN‐γ from activated T cells.^[^ ^179^ ^]^ ICANS affects 5–12% of patients and is linked to disruption of the blood‐brain barrier by inflammatory cytokines.^[^ ^180^ ^]^ 4) Complex manufacturing and high costs. The production of autologous CAR‐T cells requires 2–4 weeks and costs ≈$400 000 per patient, with ≈30% of samples failing quality control due to T cell dysfunction.^[^ ^181^ ^]^

The convergence of basic and clinical research paradigms, coupled with the integration of cross‐disciplinary technologies and international collaborative networks, has emerged as a transformative force in the development of targeted therapies. This synergistic approach leverages collective expertise and technological strengths from diverse fields, facilitating the efficient screening and validation of novel targets and thereby accelerating the translational pipeline from preclinical discovery to clinical application. Collectively, these integrated strategies establish a comprehensive framework for target discovery and validation, significantly enhancing the potential for improved therapeutic outcomes in the management of solid tumors (Figure 5C).

Emerging challenges in the hypoxic TME include metabolic exhaustion of CAR‐T cells, characterized by diminished mitochondrial function and ATP production, as well as epigenetic reprogramming that contributes to T cell exhaustion, such as the hypermethylation of the TCF7 locus observed in exhausted CAR‐T cells.^[^ ^182^, ^183^ ^]^ Additionally, the issue of allogeneic CAR‐T rejection remains unresolved.^[^ ^180^ ^]^

Recent research analyses suggest that CAR‐T cell therapy is on the verge of redefining the paradigm of cancer treatment by integrating advanced insights from immunology, synthetic biology, and clinical medicine.^[^ ^184^ ^]^ Although CAR‐T projects utilizing synthetic biology have not yet been fully realized in clinical applications, numerous projects are currently in research and development, as well as phase I/II clinical trials. It is anticipated that these innovations will significantly enhance the application of CAR‐T therapy in solid tumors in the future.

Innovative engineering of CAR‐T cells through metabolic reprogramming strategies—such as enhancing lipolysis via PPAR‐γ overexpression—combined with modifications of tumor‐homing receptors, exemplified by CXCR2‐targeted localization in fibrotic tumors, has shown breakthrough therapeutic potential in preclinical investigations.^[^ ^185^, ^186^, ^187^, ^188^ ^]^ In terms of safety, closed‐loop control systems have emerged as crucial strategies for mitigating CRS and neurotoxicity.

Technological convergence is driving the next generation of cell therapy innovation. CRISPR‐Cas12b multiplex editing can simultaneously knock out inhibitory receptors^[^ ^189^ ^]^ and insert microenvironment‐responsive cytokine genes, such as hypoxia‐inducible IL‐12.^[^ ^190^ ^]^ Additionally, artificial intelligence tools that leverage single‐cell omics data are accelerating CAR epitope design, optimizing binding kinetics, and reducing off‐target effects. Beyond traditional T cells, CAR‐M and CAR‐NK serve as versatile platforms capable of infiltrating ‘immune‐cold tumors’ and mediating non‐antigen dependent phagocytosis.^[^ ^164^, ^191^ ^]^ Notably, cell therapies are extending beyond oncology to address a variety of medical conditions, including autoimmune diseases exemplified by CD19‐CAR Tregs for multiple sclerosis and chronic infections represented by CCR5‐modified CAR‐T therapies targeting HIV reservoir clearance.^[^ ^192^ ^]^ This highlights the broad applicability of cell‐type‐specific programming.

Clinical translation hinges on overcoming critical bottlenecks: scalable manufacturing remains a central challenge. Decentralized production models, based on mRNA‐encoded transient CAR expression or lyophilized cell formulations, hold promise for enhancing global accessibility. Regulatory frameworks must adapt to the unique risks associated with engineered cells, such as long‐term genomic instability resulting from viral vector integration. Collaboration among academia, industry, and regulatory bodies should focus on standardizing safety monitoring with particular emphasis on implementing cutting‐edge technologies such as droplet digital PCR for precise tracking of vector insertion loci and spatial transcriptomics to evaluate dynamic changes in TME remodeling. Ethical considerations necessitate addressing equitable access and the ecological impact of long‐term persistence of engineered cells. In clinical practice, bridging therapy has become a crucial strategy in hematological malignancies for patients awaiting CAR‐T therapy, using corticosteroids, rituximab, and other agents to control disease progression and optimize patient status^[^ ^193^, ^194^, ^195^, ^196^ ^]^ Although the application of bridging therapy in solid tumors is limited, its success and theoretical value in hematological malignancies suggest that in‐depth exploration of this strategy may provide a new therapeutic window for solid tumor patients, reducing the risk of disease progression and enhancing CAR‐T efficacy, representing a potential new clinical translation paradigm.

Looking ahead, cell therapies will evolve from static interventions to dynamic “living drugs”: integrated biosensor circuits can autonomously regulate CAR signaling intensity based on real‐time biomarker feedback. Personalized validation using patient‐derived organoids holds the promise of achieving “context‐aware therapy” through precise calibration of the immune system. By integrating ethical AI and sustainable principles into therapeutic design, along with CRISPR‐mediated gene editing for universal CAR‐T production, synthetic biology‐driven cytokine armoring, and clinical strategies for TME modulation, cell therapies in the next decade will not only address refractory diseases but also establish a new paradigm of bioeconomy centered on programmable immunity. This vision relies on the collaborative advancement of multidisciplinary technologies, such as AI‐optimized protein design to enhance CAR binding kinetics, synthetic circuits for context‐aware signaling, and surgical‐assisted delivery to overcome the barriers posed by solid tumors, all while adhering to ethical frameworks that ensure equitable access and ecological safety.

## Conflict of Interest

The authors declare no conflict of interest.

## Author Contributions

Y.C. and R.R. contributed equally to this work. Y.C. performed conceptualization and wrote the final manuscript. R.R. performed visualization, figure preparation. Y.Z., R.S., H.S., and L.Y. performed the literature review. H.Y. wrote, reviewed, and edited the final manuscript. Y.L. performed supervision, wrote, reviewed, and edited the final manuscript. All authors read and approved the final manuscript.

## Supporting information



Supporting Information
